# Fetuin-A Concentration in the Perinatal Period and Maternal BMI Dynamics During Pregnancy, Labor, and Early Postpartum: Is ΔBMI a Parameter Worth Considering?

**DOI:** 10.3390/jcm14196782

**Published:** 2025-09-25

**Authors:** Aleksandra Obuchowska-Standyło, Żaneta Kimber-Trojnar, Monika Czuba, Katarzyna Trojnar, Bożena Leszczyńska-Gorzelak

**Affiliations:** 1 Chair and Department of Obstetrics and Perinatology, Medical University of Lublin, 20-090 Lublin, Poland; bozena.leszczynska-gorzelak@umlub.edu.pl; 2 Department of Clinical Genetics, Medical University of Lublin, 20-080 Lublin, Poland; monika.czuba@umlub.edu.pl; 3 Student’s Scientific Association at the Chair and Department of Obstetrics and Perinatology, Medical Faculty, Medical University of Lublin, 20-090 Lublin, Poland; katarzynaatrojnar@gmail.com

**Keywords:** fetuin-A, bioelectrical impedance analysis, gestational weight gain, pregnancy, postpartum

## Abstract

**Background/Objectives**: Fetuin-A is a multifunctional glycoprotein involved in metabolic and inflammatory regulation. Although its role in insulin resistance, type 2 diabetes, and cardiovascular disease is well recognized, its relationship with pregnancy-related body mass changes remains unclear. This study aimed to explore associations between maternal BMI dynamics during and shortly after pregnancy and serum fetuin-A concentrations. **Methods:** Fifty-five healthy Caucasian women with term singleton pregnancies were enrolled. BMI was recorded at three time points: pre-pregnancy, before delivery, and 48 h postpartum. Based on ΔBMI (postpartum minus pre-pregnancy BMI), participants were divided into two groups: ΔBMI ≤ 1 kg/m^2^ (*n* = 32) and ΔBMI > 1 kg/m^2^ (*n* = 23). Serum fetuin-A levels were measured before delivery and postpartum using ELISA. Additional laboratory parameters and body composition were assessed postpartum via standard tests and bioelectrical impedance analysis (BIA). **Results:** No significant differences were found between groups in BMI at any single time point or in laboratory or BIA-derived parameters. However, all three BMI change indices (ΔBMI_gestational, ΔBMI_puerperal, and ΔBMI) differed significantly between groups. Fetuin-A concentrations did not differ significantly between groups. Importantly, fetuin-A levels decreased significantly after delivery in both groups, suggesting a potential role of the placenta in its regulation. A significant correlation was observed between pre-delivery fetuin-A and postpartum uric acid in Group ΔBMI > 1 kg/m^2^ (*p* = 0.016), indicating a possible link in women with greater gestational weight gain. **Conclusions:** While fetuin-A was not directly associated with BMI changes, its peripartum dynamics and correlation with uric acid may reflect underlying metabolic-inflammation pathways. ΔBMI indices may offer a more individualized measure of weight dynamics in pregnancy research.

## 1. Introduction

Fetuin-A was first identified in 1944 in the blood of a newborn calf [1]. Subsequent studies in the early 1960s led to its isolation in human plasma, and it became known as α2-Heremans-Schmid glycoprotein (AHSG) [2]. Fetuin-A is a heterodimeric plasma glycoprotein composed of an A-chain (282 amino acids) and a B-chain (27 amino acids), connected by a single disulfide bond [2]. While the adult liver remains the principal site of fetuin-A synthesis, in the fetal period this protein is abundantly expressed in various tissues, including the liver, kidneys, brain, choroid plexus, skin, and gastrointestinal tract. Recent studies also indicate that pancreatic β-cells may contribute to fetuin-A secretion, where it appears to modulate local immune responses and promote β-cell dysfunction [3,4,5]. Moreover, expression of fetuin-A has been demonstrated in adipocytes and monocytes/macrophages, highlighting its diverse tissue origin and potential roles in both systemic and tissue-specific metabolic and inflammatory pathways [6].

Fetuin-A is widely recognized as a multifunctional molecule involved in numerous metabolic processes, including energy expenditure, appetite regulation, insulin resistance (IR), and adipogenesis [7,8]. Furthermore, fetuin-A functions as an inhibitor of ectopic calcification and protease activity, while also exhibiting dual immunological roles: it may act as a pro-inflammatory mediator or as an anti-inflammatory agent, depending on the physiological context [8,9,10,11,12]. It has been identified as both an atherogenic and adipogenic factor, highlighting its complex, context-dependent role in metabolic regulation and cardiovascular health [13]. The role of fetuin-A in the development of various clinical conditions such as IR, type 2 diabetes (T2DM), metabolic dysfunction–associated steatotic liver disease (MASLD), cardiovascular diseases (CVD), tumors and brain disorders is also confirmed [13,14,15,16,17,18,19,20]. Inflammation affects fetuin-A secretion in a dual manner. While chronic low-grade inflammation, such as in metabolic syndrome, increases its levels, acute inflammation suppresses its hepatic synthesis, as fetuin-A is a negative acute-phase protein [21].

Fetuin-A levels in healthy non-pregnant women fall within a physiologically defined range; however, studies suggest that its concentration is markedly higher in fetal circulation, with estimates indicating a several-fold increase compared to adult values [22]. According to findings from a longitudinal cohort study by Häusler et al., serum fetuin-A levels peak during mid-gestation (approximately between weeks 23 and 30), gradually decline toward term, and subsequently stabilize throughout childhood until puberty [23]. Functionally, fetuin-A has been identified as a cofactor—acting in concert with spermidine—in the suppression of tumor necrosis factor α (TNF-α) expression, a key pro-inflammatory cytokine. This immunomodulatory capacity is believed to contribute to the establishment of maternal immune tolerance toward the semi-allogeneic fetus [24,25,26].

Fetuin-A secretion is modulated by a wide range of physiological, metabolic, and pathological factors. Among the most influential is the organism’s metabolic state. Elevated circulating fetuin-A levels have consistently been observed in conditions such as obesity, IR, T2DM, and polycystic ovary syndrome (PCOS), all of which are closely linked to excessive visceral adiposity [22]. During normal pregnancy, circulating fetuin-A concentrations increase progressively, reflecting maternal metabolic adaptation. However, recent evidence suggests that fetuin-A levels are significantly higher in pregnancies complicated by metabolic disorders such as gestational diabetes mellitus (GDM) [27,28]. To date, no studies have investigated the association between pregnancy-related weight gain, postpartum weight loss, and circulating fetuin-A concentrations.

## 2. Materials and Methods

The study included 55 Caucasian women with singleton term pregnancies who delivered at the Chair and Department of Obstetrics and Perinatology in Lublin. Eligible participants met the following criteria: age between 18 and 43 years, singleton pregnancy, and term delivery (between 38 and 41 weeks of gestation). All included patients were free from chronic or gestational diseases, which significantly limited the recruitment process.

Exclusion criteria comprised multiple pregnancy, a history of endocrine disorders, the use of medications affecting metabolic parameters, and contraindications to bioelectrical impedance analysis (BIA), including current or past oncological treatment, diagnosed malignancies, epilepsy, or the presence of a cardiac pacemaker.

Body mass index (BMI) was recorded at three time points: before pregnancy or early pregnancy (calculated based on the pregnancy card and physician’s entry), on the day of delivery (prior to labor), and 48 h postpartum. Based on these values, three BMI change indices were calculated:

- ΔBMI_gestational (ΔBMI_g)—gestational BMI change defined as the difference between BMI on the day of delivery and pre-pregnancy BMI;
- ΔBMI_puerperal (ΔBMI_p)—puerperal BMI change defined as the difference between BMI on the day of delivery and BMI 48 h postpartum;
- ΔBMI—total BMI change defined as the difference between BMI on the second postpartum day and pre-pregnancy BMI.

Participants were stratified into two groups based on ΔBMI: the study group (ΔBMI > 1 kg/m^2^; *n* = 23) included women with a greater overall BMI change, whereas the control group (ΔBMI ≤ 1 kg/m^2^; *n* = 32) comprised women with minimal or negative BMI change. The threshold of ΔBMI > 1 kg/m^2^ was selected because this degree of change is considered clinically meaningful in the context of maternal weight dynamics and may be associated with differences in both metabolic and obstetric outcomes. This choice was also motivated by exploratory considerations derived from the distribution of our dataset. To date, the literature has most commonly applied criteria for adequate and excessive gestational weight gain expressed in kilograms, stratified by pre-pregnancy BMI. However, weight gain expressed in kilograms does not account for maternal height, which carries important clinical implications. By normalizing weight for height, BMI serves as a more objective indicator and facilitates clinically relevant comparisons between women of different heights. Moreover, normative patterns of postpartum weight reduction in relation to gestational weight gain have not been described. Therefore, there is a need to identify indicators that more accurately reflect the metabolic status of pregnant women.

Venous blood samples were collected before delivery and 48 h postpartum. Biochemical analyses were performed after a 6 h fasting in the early post-partum period in a certified laboratory and included fasting glucose, insulin, insulin resistance index (HOMA-IR), homocysteine, high-density lipoprotein (HDL) cholesterol, low-density lipoprotein (LDL) cholesterol, triglycerides, uric acid, ferritin, creatinine, and complete blood count.

Maternal serum concentrations of fetuin-A were measured at two time points—before delivery and 48 h postpartum—using the Human Fetuin A Quantikine ELISA Kit (DFTA00, R&D Systems) kit via traditional enzyme-linked immunosorbent assay (ELISA). All analyses were performed according to the manufacturer’s instructions.

Maternal body composition and hydration status were evaluated 48 h postpartum using BIA, performed with the Body Composition Monitor (BCM; Fresenius Medical Care). This method enabled non-invasive assessment of fat mass, lean body mass, and fluid compartments in the early puerperium.

The study was approved by the Bioethics Committee of the Medical University of Lublin (approval no. KE-0254/61/2020), and written informed consent was obtained from all participants prior to enrolment, in accordance with the ethical standards for research involving human subjects.

Statistical analysis was performed using Statistica software (v13.3, StatSoft, Tulsa, OK, USA). Data expressed on a quantitative scale were presented as the mean, standard deviation (SD), interquartile range (IQR), minimum, and maximum values. The consistency of variable distributions with the normal distribution was assessed using the Shapiro–Wilk test. Student’s *t*-test for independent samples or the Mann–Whitney U test was used to evaluate differences between two groups. The Wilcoxon signed-rank test was used to assess changes in the studied parameters over time. Relationships between variables were evaluated using Spearman’s rank correlation coefficient. Results were considered statistically significant at *p* < 0.05.

## 3. Results

No statistically significant differences were observed between the two groups in BMI measured at any of the three time points (pre-pregnancy, before delivery, and 48 h postpartum). Similarly, there were no significant differences in other anthropometric, laboratory, or BIA-derived parameters. A detailed comparison of these variables is presented in Table 1.

Statistically significant differences were observed between the groups across all three BMI change indices—ΔBMI_gestational (ΔBMI_g), ΔBMI_puerperal (ΔBMI_p), and ΔBMI, as presented in Table 2.

During pregnancy, participants in the study group exhibited a significantly greater gestational BMI increase (ΔBMI_g) compared to the control group (*p* < 0.001).

Similarly, ΔBMI_p (reflecting the difference between BMI at delivery and 48 h postpartum) was significantly higher in the control group than in the study group (*p* < 0.001).

The total BMI change from pre-pregnancy to postpartum (ΔBMI) also differed markedly between groups. The study group showed a mean increase, whereas the control group demonstrated a mean decrease (*p* < 0.001).

**Figure 1:**
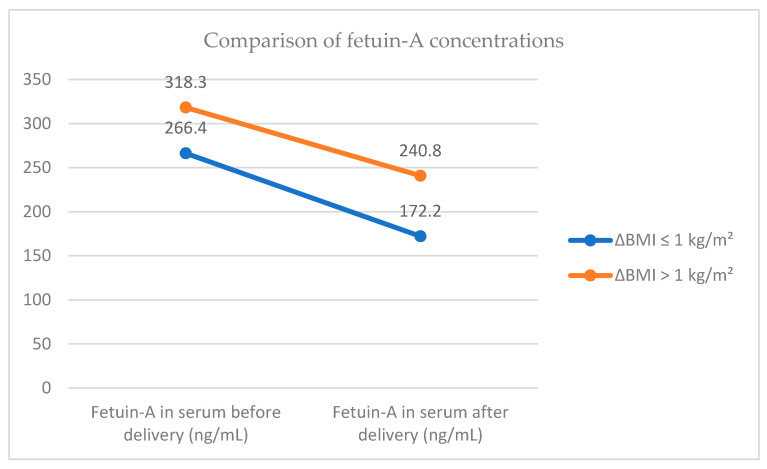
Comparison of fetuin-A concentrations.

In both groups, we observed a statistically significant decrease in fetuin-A concentrations after delivery compared to pre-delivery levels. That is, fetuin-A levels decreased significantly postpartum in both Group ΔBMI ≤ 1 kg/m^2^, (*p* < 0.001) and Group ΔBMI > 1 kg/m^2^, (*p* < 0.001) (Figure 1).

No statistically significant differences in fetuin-A concentrations were observed between the two groups, either before or after delivery. Likewise, no significant intergroup differences were found in the change in fetuin-A levels (i.e., the difference between the measurement at 48 h postpartum and the measurement before delivery) (Table 3).

Correlations between pre-delivery and postpartum serum fetuin-A concentrations and selected biochemical and biophysical parameters are presented in Table 4 and Table 5, respectively.

Among the analyzed biochemical and biophysical parameters, a statistically significant correlation was found between pre-delivery fetuin-A concentration and uric acid levels in Group ΔBMI > 1 kg/m^2^ (*p* = 0.016). No such correlation was observed in Group ΔBMI ≤ 1 kg/m^2^. Postpartum serum fetuin-A concentrations did not show any significant correlations in either study group (Table 5).

Correlations between pre-delivery and postpartum serum fetuin-A concentrations and three BMI delta indices—ΔBMI_g, ΔBMI_p, and ΔBMI are presented in Table 6 and Table 7, respectively. No statistically significant results were found between the tested parameters.

## 4. Discussion

Weight gain in pregnancy is a physiological process responding to fetal development, encompassing changes in maternal body composition as well as the weight of the fetus, placenta, and amniotic fluid [29]. It is also an important predictor of healthy pregnancy and delivery outcomes [30]. Although established guidelines exist for healthy gestational weight gain based on pre-pregnancy BMI [31], supported by dietary and physical activity recommendations, an increasing number of women experience excessive gestational weight gain (EGWG), which negatively impacts the metabolic health of both mother and offspring.

In this study, we opted to analyze BMI changes expressed in kg/m^2^ rather than the conventional measure of gestational weight gain in kilograms. This approach offers a more precise comparison by normalizing for individual differences in height, which is an important factor when assessing body mass dynamics. Significant differences were identified between Group ΔBMI ≤ 1 kg/m^2^ and Group ΔBMI > 1 kg/m^2^ across all three examined BMI change indices: the increase in BMI during pregnancy (ΔBMI_g), the decrease in BMI in the early postpartum period (ΔBMI_p), and the total BMI change from pre-pregnancy to 48 h postpartum (ΔBMI). These indices provide a comprehensive evaluation of maternal body mass fluctuations around the peripartum period.

The total BMI change index (ΔBMI) is particularly valuable as it reflects the overall gestational BMI gain while reducing potential confounding effects caused by physiological changes inherent to pregnancy, such as fetal growth, amniotic fluid volume, placental mass, expansion of maternal blood volume, and fluid retention. This makes ΔBMI a more individualized and reliable marker of maternal metabolic changes. It is important to note that pre-pregnancy BMI values were similar between the groups, indicating that the observed differences in BMI dynamics were not attributable to baseline body mass but rather to distinct patterns of weight change during and after pregnancy.

The ΔBMI_g index accounts for pregnancy-related confounding factors such as fetal mass, amniotic fluid volume, placental weight, increased maternal blood and fluid volume, adipose tissue accumulation, as well as uterine and breast enlargement. This index was significantly lower in the control group compared to the study group, indicating greater gestational BMI gain in the latter. Despite these differences in gestational BMI increase, the study population was limited to women with term singleton pregnancies, and cases with complications such as oligohydramnios or polyhydramnios were excluded to further reduce potential confounders. Moreover, neonatal birth weight did not differ significantly between groups, nor were there significant differences in maternal body composition assessed postpartum.

Interestingly, although the control group exhibited a smaller increase in gestational BMI, it experienced a significantly greater reduction in BMI during the early postpartum period compared to the study group. This suggests distinct patterns of maternal weight change between the groups in both pregnancy and the immediate postpartum phase.

In both groups of our study–women with ΔBMI ≤ 1 kg/ m^2^ and those with ΔBMI > 1 kg/ m^2^–we observed a statistically significant decrease in fetuin-A levels 48 h postpartum compared to levels measured immediately before delivery. To our knowledge, fetuin-A concentrations have not previously been compared within such a short peripartum interval (i.e., over a span of two days). This novel observation may suggest a possible role of the placenta in fetuin-A dynamics.

A study by Wang et al. (2019) demonstrated increased placental fetuin-A mRNA and protein expression in women with GDM compared to healthy pregnant women, suggesting that fetuin-A is produced in the placenta and that its levels may vary in pathological states [32]. This was further confirmed by Šimják et al. (2018), who also showed placental fetuin-A mRNA expression in both healthy and GDM pregnancies, supporting the concept of local synthesis within the placenta [27].

Several studies have examined fetuin-A fluctuations during pregnancy. In a study by Šimják et al. (2018), fetuin-A levels were shown to increase throughout pregnancy and decrease after delivery, regardless of GDM status [27]. Concentrations were measured at three time points: between 28 and 32 weeks, 36 and 38 weeks of gestation, and 6 and 12 months postpartum. Healthy pregnant women had higher fetuin-A levels during pregnancy than non-pregnant controls, though levels significantly declined by 6–12 months postpartum. No significant differences were found between healthy and GDM pregnancies in fetuin-A levels during pregnancy [27].

Similar findings were reported by Iyidir et al. (2015), who observed higher fetuin-A levels during pregnancy in women with GDM, with a significant postpartum decrease [28]. However, the study involved only 26 women, with fetuin-A assessed between 24 and 28 weeks and then in 18 women at 3 months postpartum [28].

In a study by Albuquerque et al. (2019), conducted in 60 low-risk pregnant women, maternal serum fetuin-A was shown to decrease significantly in the days preceding delivery [33]. Interestingly, fetuin-A levels were found to be higher more than 6 weeks postpartum compared to levels at delivery–a finding differing from both previous studies (measured at later postpartum points) and from our results (measured within 48 h postpartum) [33].

We did not observe any significant correlation between fetuin-A levels, measured either immediately before delivery or 48 h postpartum, and any parameters of glucose or lipid metabolism. Our results align with previous studies in healthy pregnant women. For example, Iyidir et al. (2015) found no significant correlations between fetuin-A and hemoglobin A1c or lipid parameters (total cholesterol and triglycerides) in healthy pregnancies [28]. Similarly, Yakout et al. (2023) reported no significant associations between fetuin-A and insulin resistance markers (e.g., insulin, HOMA-IR) in healthy pregnant women [34]. This supports the idea that fetuin-A may not play a direct role in glucose or lipid metabolism during uncomplicated pregnancies.

In our study, fetuin-A levels measured both before delivery and 48 h postpartum did not correlate with any BMI value (pre-pregnancy, at delivery, or postpartum), nor with any of the calculated BMI change indices (ΔBMI_g, ΔBMI_p, or ΔBMI_t). Additionally, none of the numerous parameters obtained via BIA at 48 h postpartum showed correlation with fetuin-A concentrations at either time point.

By contrast, Farhan et al. (2012) reported a strong positive correlation between fetuin-A and BMI in women with a history of GDM in the postpartum period (r = 0.90, *p* < 0.0001) [35]. Notably, this study did not find any differences in fetuin-A between women with prior GDM and healthy controls during pregnancy, nor did it observe an association between BMI and fetuin-A during gestation [35]. In line with our findings, Šimják et al. (2018) also reported no association between fetuin-A and BMI in pregnant women [27].

Comparison of our findings regarding the lack of correlation between fetuin-A levels and BIA parameters in the early postpartum period with other studies is not feasible, as no previous studies have examined such relationships—not only in the early puerperium but, to our knowledge, at any point across the human lifespan. Most research to date has focused on associations with BMI and other anthropometric indices. For instance, a study in non-pregnant women with PCOS by Kulik-Kupka et al. (2022) found no significant correlation between fetuin-A and BMI or other anthropometric indices such as BAI (Body Adiposity Index), VAI (Visceral Adiposity Index), LAP (Lipid Accumulation Product), or ABSI (A Body Shape Index) [36].

In our study, a significant positive correlation between serum fetuin-A concentrations before delivery and serum uric acid concentration was observed exclusively in Group ΔBMI > 1 kg/m^2^ (r = 0.49, *p* = 0.015). This correlation was not found in Group ΔBMI ≤ 1 kg/m^2^, with minimal or negative BMI changes. Both fetuin-A and uric acid are biomarkers associated with metabolic dysfunction and inflammation; however, their direct relationship remains incompletely understood. Emerging evidence suggests they may be linked through shared metabolic and inflammatory pathways. In patients with chronic kidney disease, fetuin-A was shown to indirectly exert significant negative effects on estimated glomerular filtration rate (eGFR) via BMI and uric acid, suggesting that increases in fetuin-A levels are associated with increases in BMI and uric acid [37]. However, the single-mediator pathway involving only fetuin-A and uric acid was not statistically significant [37]. Additionally in a study on non-alcoholic fatty liver disease (NAFLD)—the former term for what is now referred to as MASLD—fetuin-A was shown to be directly associated with BMI, triglycerides, and uric acid [38]. High uric acid concentrations have pro-inflammatory effects and may activate inflammasomes, potentially increasing production of fetuin-A. Both molecules are also associated with IR and T2DM [7,39]—uric acid contributes to metabolic dysfunction, which may lead to increased fetuin-A levels. Lipolysis associated with excess visceral adipose tissue causes excessive release of free fatty acids (FFAs) into the bloodstream, promoting chronic subclinical inflammation and exacerbating IR in target tissues [6,8,40,41,42,43,44]. Elevated blood glucose and FFAs may stimulate fetuin-A synthesis and secretion while concurrently increasing uric acid levels, potentially creating an indirect feedback loop linking these metabolic factors [45]. It was found that the concentration of uric acid correlates positively with triglycerides and negatively with HDL cholesterol, and its level is an independent risk factor for the development of metabolic syndrome [46,47]. In addition, hyperinsulinemia causes a decrease in the excretion of uric acid in the urine through the effect of insulin on the urinary tubules, which leads to hyperuricemia [48]. Moreover, fetuin-A and uric acid are implicated in CVD pathogenesis through inflammation and endothelial dysfunction [20,49,50]. Lower plasma fetuin-A levels associate with higher mortality risk in coronary artery disease patients, independent of traditional risk factors [51]. During pregnancy, both biomarkers (fetuin-A and uric acid) may influence the placental microenvironment contribute to complications like preeclampsia [52,53,54,55]. Previous studies have demonstrated that maternal plasma concentrations of fetuin-A at the time of preterm PE diagnosis are lower compared to controls with uncomplicated pregnancies [52,53,56]. However, such differences were not observed in patients with term PE [52]. Moreover, fetuin-A levels were found to correlate with the concentrations of other negative acute-phase reactants, such as albumin and transferrin, in patients with PE [52], whereas CRP levels were significantly higher in the PE group [53]. These findings suggest that fetuin-A concentration may reflect the presence of intravascular inflammation.

Although the interaction between fetuin-A and uric acid remains incompletely understood, elevated pre-delivery fetuin-A levels in women with greater gestational BMI changes may reflect increased inflammatory signaling linked to higher postpartum uric acid. This suggests a dynamic interplay between metabolic stress and inflammation in the peripartum period, potentially contributing to postpartum metabolic adaptations or risks. Future research could explore whether interventions targeting uric acid or fetuin-A pathways may mitigate adverse metabolic outcomes in this population.

Limitations of our study include a relatively small sample size, although this was a consequence of stringent inclusion and exclusion criteria—participants were free from chronic or gestational disorders, ensuring a highly homogeneous cohort. The only factor differentiating the two groups was gestational weight gain (ΔBMI_t), while all other variables—age, singleton term pregnancy, and absence of other health conditions—were essentially uniform across groups. This deliberate homogeneity enhances internal validity but may restrict external generalizability, as findings may not apply to broader populations such as older women, multiple gestations, or individuals with comorbidities.

## 5. Conclusions

Although we did not observe significant correlations between serum fetuin-A concentrations (pre- or postpartum) and any BMI value or BMI change indices (ΔBMI_g, ΔBMI_p, ΔBMI), an interesting association emerged in women with greater total BMI increase during pregnancy (Group ΔBMI > 1 kg/ m^2^), where a significant positive correlation was found between pre-delivery fetuin-A and postpartum uric acid levels. This suggests a possible interaction between fetuin-A and uric acid via shared metabolic or inflammatory pathways, particularly in women with substantial gestational weight gain.

Our findings support the role of serum uric acid as a more direct marker of postpartum metabolic status than fetuin-A in this specific population. However, the observed association between fetuin-A and uric acid in women with high ΔBMI may point to an indirect pathway linking inflammatory and metabolic adaptations during pregnancy and the early puerperium.

Importantly, we propose that BMI-based indices of weight change (ΔBMI) may offer a more individualized and accurate measure of gestational and postpartum weight dynamics compared to traditional weight gain metrics. Despite similar mean pre-pregnancy BMI in both groups, women in the control group demonstrated a lower ΔBMI_g (gestational gain), greater ΔBMI_p (postpartum reduction), and a negative ΔBMI (total change)—highlighting distinct patterns of metabolic adaptation not captured by absolute weight changes. These results suggest that ΔBMI metrics may be more informative for studying maternal metabolic trajectories, and future research should consider their broader application in perinatal metabolic studies.

Notably, we observed a statistically significant decrease in serum fetuin-A levels within 48 h after delivery compared to levels measured immediately prior to birth in both study groups. To our knowledge, this is the first study to report such a rapid peripartum decline in fetuin-A concentrations. This finding may reflect the role of the placenta as a source of fetuin-A during pregnancy, and its removal at delivery could partially explain the observed dynamics. However, these findings require confirmation in larger study cohorts, and it would be highly valuable for future research to incorporate a longer follow-up period during the postpartum phase. Future research is warranted to explore the placental contribution to maternal circulating fetuin-A and its metabolic relevance in the immediate postpartum period.

In addition, in our previous studies, our research team investigated the concentrations of various adipokines and cytokines during the perinatal period and examined their associations with BIA parameters [30,57,58,59,60,61,62,63,64]. To the best of our knowledge, this is the first study to investigate the relationship between fetuin-A and BIA. Furthermore, future studies should also focus on other glycoproteins and their associations with BMI changes during pregnancy and the early postpartum period, in order to gain a better understanding of the pathophysiological processes occurring during this time.

## Figures and Tables

**Table 1:** Comparison of baseline characteristics between groups.

Variables		Group ΔBMI ≤ 1 kg/m^2^ *n* = 32	Group ΔBMI > 1 kg/m^2^ *n* = 23	*p*
Age (years)	Mean (SD)	32.9 (4.2)	32.0 (5.7)	0.49 #
Gestational age at delivery (weeks)	Mean (SD)	38.7 (1.2)	38.4 (1.2)	0.38 ^
Pre-pregnancy BMI (kg/m^2^)	Mean (SD)	28.9 (7.2)	27.1 (7.2)	0.18 ^
BMI on the day of delivery (kg/m^2^)	Mean (SD)	31.9 (6.6)	32.0 (7.7)	0.78 ^
BMI on the second day of postpartum period (kg/m^2^)	Mean (SD)	27.9 (7.1)	29.9 (7.7)	0.32 ^
Neonatal birth weight (g)	Mean (SD)	3402 (415)	3429 (495)	0.83 #
High-density lipoprotein (mg/dL)	Mean (SD)	73.1 (16.1)	72.3 (15.7)	0.86 #
Low-density lipoprotein (mg/dL)	Mean (SD)	140.5 (28.7)	129.3 (26.8)	0.15 #
Triglycerides (mg/dL)	Mean (SD)	174.3 (26.7)	167.1 (28.7)	0.34 #
Uric acid (mg/dL)	Mean (SD)	5.1 (1.1)	5.2 (1.0)	0.85 ^
Ferritin (µg/L)	Mean (SD)	18.1 (14.4)	16.6 (11.5)	0.56 ^
Homocysteine (µmol/L)	Mean (SD)	10.8 (3.3)	10.3 (2.8)	0.53 ^
Total body water (%)	Mean (SD)	35.9 (5.8)	37.3 (6.3)	0.39 #
Extracellular water to intracellular water index (E/I)	Mean (SD)	0.9 (0.2)	0.9 (0.1)	0.37 ^
Lean tissue index (kg/m^2^)	Mean (SD)	13.5 (3.1)	13.0 (1.7)	0.61 ^
Fat tissue index (kg/m^2^)	Mean (SD)	14.3 (7.7)	16.8 (7.9)	0.24 ^
Body cell mass (kg)	Mean (SD)	20.1 (6.3)	19.7 (4.4)	0.93 ^

SD—standard deviation; #—Student’s *t*-test; ^—Mann–Whitney test.

**Table 2:** Comparison of BMI changes between groups.

		Group ΔBMI ≤ 1 kg/m^2^ *n* = 32	Group ΔBMI > 1 kg/m^2^ *n* = 23	*p*
ΔBMI_g (kg/m^2^)	Mean (SD)	2.9 (1.2)	4.9 (1.7)	**<0.001**
ΔBMI_p (kg/m^2^)	Mean (SD)	3.9 (1.5)	2.2 (0.9)	**<0.001**
ΔBMI (kg/m^2^)	Mean (SD)	−0.9 (1.3)	2.7 (1.7)	**<0.001**

SD—standard deviation; *p*—Mann–Whitney test; ΔBMI—total BMI change defined as the difference between BMI on the second postpartum day and pre-pregnancy BMI; ΔBMI_g—ΔBMI_gestational—gestational BMI change defined as the difference between BMI on the day of delivery and pre-pregnancy BMI; ΔBMI_p—ΔBMI_puerperal-puerperal BMI change defined as the difference between BMI on the day of delivery and BMI 48 h postpartum.

**Table 3:** Comparison of fetuin-A concentrations before and after delivery, and their changes between groups.

		Group ΔBMI ≤ 1 kg/m^2^ *n* = 32	Group ΔBMI > 1 kg/m^2^ *n* = 23	*p*
Fetuin-A in serum before delivery (ng/mL)	Mean (SD)	266.4 (102.2)	318.3 (199.4)	0.61
Fetuin-A in serum after delivery (ng/mL)	Mean (SD)	172.2 (63.7)	240.8 (191.4)	0.25
Δ fetuin-A (ng/mL)	Mean (SD)	−94.2 (76.5)	−77.5 (62.2)	0.58

SD—standard deviation; *p*—Mann–Whitney test; Δ fetuin-A—difference between fetuin A concentration in serum before and after delivery.

**Table 4:** Correlations between pre-delivery serum fetuin-A concentrations and selected biochemical and biophysical parameters.

Parameter	Group ΔBMI ≤ 1 kg/m^2^ *n* = 32	Group ΔBMI > 1 kg/m^2^ *n* = 23
Insulin resistance index (HOMA-IR)	r = 0.09, *p* = 0.64	r = 0.12, *p* = 0.58
Homocysteine (µmol/L)	r = 0.04, *p* = 0.84	r = 0.13, *p* = 0.55
High-density lipoprotein (mg/dL)	r = 0.19, *p* = 0.3	r = 0.22, *p* = 0.33
Low-density lipoprotein (mg/dL)	r = 0.22, *p* = 0.22	r = −0.09, *p* = 0.66
Triglycerides (mg/dL)	r = −0.01, *p* = 0.96	r = −0.00, *p* = 0.99
Uric acid (mg/dL)	r = 0.17, *p* = 0.34	r = 0.5, ***p* = 0.02**
Ferritin (µg/L)	r = 0.20, *p* = 0.26	r = 0.18, *p* = 0.41
Total body water (%)	r = 0.11, *p* = 0.55	r = 0.16, *p* = 0.47
Extracellular water to intracellular water index (E/I)	r = 0.28, *p* = 0.13	r = −0.17, *p* = 0.44
Lean tissue index (kg/m^2^)	r = −0.12, *p* = 0.53	r = 0.27, *p* = 0.21
Fat tissue index (kg/m^2^)	r = −0.03, *p* = 0.89	r = 0.04, *p* = 0.88
Body cell mass (kg)	r = −0.09, *p* = 0.63	r = 0.1, *p* = 0.65

r—Spearman’s correlation coefficient.

**Table 5:** Correlations between postpartum serum fetuin-A concentrations and selected biochemical and biophysical parameters.

Parameter	Group ΔBMI ≤ 1 kg/m^2^ *n* = 32	Group ΔBMI > 1 kg/m^2^ *n* = 23
Insulin resistance index (HOMA-IR)	r = 0.19, *p* = 0.29	r = 0.14, *p* = 0.54
Homocysteine (µmol/L)	r = −0.00, *p* = 1.0	r = 0.03, *p* = 0.88
High-density lipoprotein (mg/dL)	r = 0.27, *p* = 0.14	r = −0.05, *p* = 0.82
Low-density lipoprotein (mg/dL)	r = 0.06, *p* = 0.74	r = −0.17, *p* = 0.44
Triglycerides (mg/dL)	r = −0.16, *p* = 0.37	r = −0.09, *p* = 0.68
Uric acid (mg/dL)	r = −0.06, *p* = 0.74	r = 0.18, *p* = 0.42
Ferritin (µg/L)	r = 0.1, *p* = 0.57	r = 0.29, *p* = 0.19
Total body water (%)	r = −0.04, *p* = 0.84	r = −0.02, *p* = 0.93
Extracellular water to intracellular water index (E/I)	r = 0.2, *p* = 0.28	r = 0.04, *p* = 0.85
Lean tissue index (kg/m^2^)	r = −0.16, *p* = 0.37	r = 0.1, *p* = 0.66
Fat tissue index (kg/m^2^)	r = 0.07, *p* = 0.69	r = 0.04, *p* = 0.85
Body cell mass (kg)	r = −0.05, *p* = 0.77	r = −0.19, *p* = 0.39

r—Spearman’s correlation coefficient.

**Table 6:** Correlations between pre-delivery serum fetuin-A concentrations and BMI delta indices.

ΔBMI	Group ΔBMI ≤ 1 kg/m^2^ *n* = 32	Group ΔBMI > 1 kg/m^2^ *n* = 23
ΔBMI_g (kg/m^2^)	r = −0.19, *p* = 0.3	r = 0.01, *p* = 0.95
ΔBMI_p (kg/m^2^)	r = 0.07, *p* = 0.71	r = 0.24, *p* = 0.27
ΔBMI (kg/m^2^)	r = −0.24, *p* = 0.19	r = −0.05, *p* = 0.84

r—Spearman’s correlation coefficient; ΔBMI_g—ΔBMI_gestational—gestational BMI change defined as the difference between BMI on the day of delivery and pre-pregnancy BMI; ΔBMI_p—ΔBMI_puerperal-puerperal BMI change defined as the difference between BMI on the day of delivery and BMI 48 h postpartum; ΔBMI—total BMI change defined as the difference between BMI on the second postpartum day and pre-pregnancy BMI.

**Table 7:** Correlations between postpartum serum fetuin-A concentrations and BMI delta indices.

ΔBMI	Group ΔBMI ≤ 1 kg/m^2^ *n* = 32	Group ΔBMI > 1 kg/m^2^ *n* = 23
ΔBMI_g (kg/m^2^)	r = −0.02, *p* = 0.93	r = 0.01, *p* = 0.95
ΔBMI_p (kg/m^2^)	r = −0.00, *p* = 1.0	r = 0.12, *p* = 0.58
ΔBMI (kg/m^2^)	r = 0.07, *p* = 0.69	r = −0.02, *p* = 0.94

r—Spearman’s correlation coefficient; ΔBMI_g—ΔBMI_gestational—gestational BMI change defined as the difference between BMI on the day of delivery and pre-pregnancy BMI; ΔBMI_p—ΔBMI_puerperal-puerperal BMI change defined as the difference between BMI on the day of delivery and BMI 48 h postpartum; ΔBMI—total BMI change defined as the difference between BMI on the second postpartum day and pre-pregnancy BMI.

## Data Availability

The data presented in this study are available on request from the corresponding author.
